# Rare case of a radiographically occult sacral lesion detected on MRI presenting with intractable back pain

**DOI:** 10.1259/bjrcr.20150002

**Published:** 2015-05-26

**Authors:** A J Degnan, C Maldjian, L Pantanowitz, J K Kofler

**Affiliations:** ^1^Department of Radiology, University of Pittsburgh Medical Center, Pittsburgh, PA, USA; ^2^University of Pittsburgh, Pittsburgh, PA, USA; ^3^Department of Pathology, University of Pittsburgh Medical Center, Pittsburgh, PA, USA

## Abstract

We report the imaging findings and histopathology of a rare case of sacral hibernoma in a female presenting with right buttock pain while sitting. The lesion was occult on radiographs and CT scan. A small, rounded right S2 lesion was hypointense on *T*_1_ weighted images and hyperintense on short tau inversion-recovery images. It demonstrated homogeneous contrast enhancement. The lesion was biopsied, and histopathology revealed an intraosseous hibernoma composed of brown fat cells. Intraosseous hibernomas are rare and demonstrate non-specific imaging findings requiring biopsy for diagnosis, although most hibernomas are incidental and asymptomatic. Initial treatment with microwave ablation and cementoplasty improved the patient’s symptoms temporarily but cementoplasty caused radicular symptoms, and eventually, cement removal, bone curettage, grafting and sacral nerve root decompression were required for symptom remission.

## Clinical presentation

**Figure 1.**
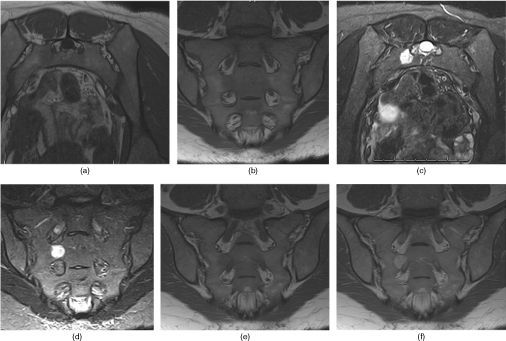
MRI of the pelvis reveals sacral lesion. Axial (a) and coronal (b) *T*_1_ weighted images of the pelvis (scan parameters: TR= 540 ms; TE= 10 ms; 640 × 640; 4-mm slice thickness) demonstrate a slightly hypointense well-demarcated rounded lesion involving the right sacrum at S2 between the anterior neural foramina of S1 and S2. This lesion was hyperintense on axial (c) and coronal (d) short tau inversion recovery (scan parameters: TR = 5400 ms; TE = 60 ms; 512 × 512; 4-mm slice thickness). Coronal pre-contrast (e) and post-contrast *T*_1_ weighted (scan parameters: TR = 700 ms; TE=10 ms; 640 × 640; 4-mm slice thickness) images (f) demonstrate homogeneous enhancement within the right sacral lesion. TE, echo time; TR, repetition time.

**Figure 2.**
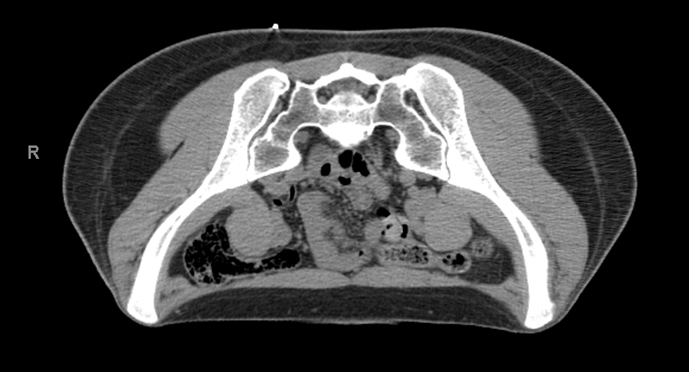
Sacral lesion is occult on CT scan. A representative axial CT image (120 kVp, 90 mA, slice thickness 3 mm) of the expected location of the intraosseous hibernoma does not demonstrate an appreciable abnormality.

A 36-year-old female presented complaining of months of worsening back and right buttock pain exacerbated by sitting. She did not respond to initial management with conservative therapy of non-steroidal anti-inflammatory medications. Radiographs of the pelvis were unremarkable. Subsequently, MRI of the lumbar spine was performed and showed a partially visible sacral lesion. Later, MRI of the pelvis demonstrated a rounded, non-destructive lesion involving the right sacrum at the S2 level. The sacral lesion was hypointense on *T*_1_ weighted images, hyperintense on *T*_2_ weighted and short tau inversion-recovery images (Figure 1) and enhanced homogeneously on contrast-enhanced images (Figure 1). CT images (Figure 2) did not demonstrate any obvious sacral lesion.

## Differential diagnosis

The differential diagnosis for this sacral lesion includes osseous haemangioma, generally with *T*_2_ hyperintensity and enhancement. In the initial evaluation of our case, haemangioma was the suspected diagnosis. An intraosseous hibernoma may also be considered as a possible entity, albeit rare, with *T*_1_ weighted hypointensity, *T*_2_ weighted hyperintensity and homogeneous enhancement. Other possible aetiologies include skeletal metastases, particularly if these are 18-fludeoxyglucose avid on positron emission tomography/CT scan, although these are not expected to be radiographically occult.^1^ One recently recognized entity capable of exhibiting a similar intraosseous appearance within the sacrum is a benign notochordal cell tumour; these benign lesions may lead to chordoma and present similar to our case as radiographically occult lesions with low *T*_1_ weighted signal and high *T*2 weighted signal and may exhibit sclerosis on CT scan.^2,3^

## Investigations

**Figure 3.**
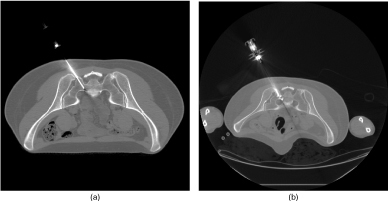
CT-guided biopsy and microwave ablation of sacral lesion. (a) Intraprocedure axial CT image (120 kVp, 90 mA, slice thickness 3 mm) of the percutaneous bone biopsy demonstrates the site of biopsy corresponded with the site of space-occupying lesion seen on MRI. Pathology confirmed intraosseous hibernoma in this location. (b) Intraprocedure axial CT image (120 kVp, 90  mA, slice thickness 3 mm) demonstrates the site of microwave ablation and cementoplasty within the same location as the probable hibernoma.

**Figure 4.**
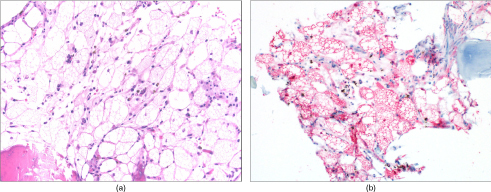
Histopathology demonstrates intraosseous hibernoma. (a) Histopathology (haematoxylin and eosin stain; magnification ×200) shows large, oval-shaped and polygonal multivacuolated brown adipose cells forming an intraosseous hibernoma. (b) Immunohistochemistry evaluation using S100 staining (red) with haematoxylin counterstain (blue) shows strong nuclear and cytoplasmic positivity for S100 in the vacuolated cells, supporting the diagnosis of intraosseous hibernoma.

**Figure 5.**
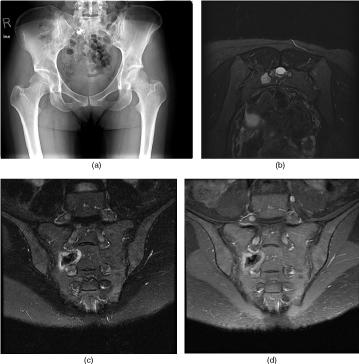
Imaging appearance after microwave ablation and cementoplasty. AP radiograph of the pelvis (a) shows ablation and cementoplasty-related changes. MRI of the pelvis with axial and coronal *T*_2_ weighted (b, c; FSE FS, TR = 3000 ms, TE = 102 ms, 512 × 512, 4-mm thickness) and post-contrast *T*_1_ weighted images (d; FSE FS + C, TR = 517 ms, TE = 11 ms, 512 × 512, 4-mm thickness) demonstrates a central hypointensity with peripheral *T*_2_ hyperintense rim and homogeneous rim of enhancement. There was also irregularity of the right S3 nerve root related to treatment changes. AP, anteroposterior; FSE FS, fast spin echo, fat saturated; FSE FS + C, post-contrast fast spin echo, fat saturated; TE, echo time; TR, repetition time.

CT-guided biopsy was performed by correlating MRI findings with bony landmarks (Figure 3). Gross pathology demonstrated red–brown bone and soft tissue. Histopathology revealed a collection of large ovoid, multivacuolated adipose cells consistent with brown fat admixed with scant hemosiderin deposits and rare chronic inflammatory cells, including scattered plasma cells (Figure 4a). These brown fat cells had displaced the adjacent normocellular bone marrow with trilineage haematopoiesis. The bone trabeculae were unremarkable. Further immunohistochemical evaluation demonstrated strong nuclear and cytoplasmic positivity for S100 in the vacuolated cells (Figure 4b), supporting a diagnosis of intraosseous hibernoma. CT-guided biopsy and microwave ablation (Figure 3), and subsequent post-treatment images (Figure 5) in the same location confirm that this biopsy was taken from the space-occupying lesion, and the gross presence of brown fat on pathology also favoured intraosseous hibernoma.

## Treatment

Initially, the patient’s sacral lesion was treated with microwave ablation and cementoplasty with initial symptom relief. A follow-up MRI of the pelvis (Figure 5) demonstrated post-treatment changes of the right sacral S2 lesion characterized by central low signal with a corona of uniform high *T*_1_ and *T*_2_ signal. There was no clear evidence of residual tumour or recurrence.

## Outcome, follow-up and discussion

Approximately 10 months after initial ablation and cementoplasty, the patient developed worsening pain in the right buttock radiating into medial and posterior thigh with associated numbness. These radicular symptoms with numbness were different from the patients initial presentation with greater severity. This pain was attributed to encroachment of cement and treatment-related changes within the right S3 nerve root suggested by earlier post-operative imaging (Figure 5). Surgery was performed with bone cement removal, curettage, decompression of the right sacral nerve roots and bone grafting of the right sacrum. Histopathological examination following this surgery did not identify residual hibernoma or recurrence. On the most recent follow-up, the patient reported pain resolution.

We describe a unique case of a symptomatic sacral intraosseous hibernoma that was occult on radiographs and CT scan. This right S2 lesion had a non-specific MRI appearance with *T*_1_ weighted hypointense, *T*_2_ weighted hyperintense and homogeneous enhancement properties. Biopsy suggested an intraosseous hibernoma. Hibernoma is a rare, benign soft-tissue tumour composed of brown fat most often manifesting as subcutaneous soft-tissue tumours involving the thigh, shoulder, back, neck, chest, arm and retroperitoneum.^4^ They typically occur as slow-growing masses that are mostly asymptomatic. This case of probable sacral intraosseous hibernoma adds to the handful of reported intraosseous cases.^1, 5–12^ To date, only 14 cases, including this one, have been reported (Table 1) in our review of published studies included on MEDLINE; the sacrum is the second most common site after the ilium.^1, 5–12^

**Table 1.** Frequency of intraosseous hibernoma by anatomical location in previously published reports^1, 5–12^

Location	Number of cases (%)
Ilium	5 (36)
Sacrum	3 (21)
Thoracic vertebrae	2 (14)
Femurs	1 (7)
Lumbar vertebrae	1 (7)
Manubrium	1 (7)
Pubic ramus	1 (7)

The sacral lesion in this case demonstrated a non-specific appearance with *T*_1_ weighted hypointensity, *T*_2_ weighted hyperintensity and homogenous enhancement, which is consistent with previously described intraosseous hibernomas. Nevertheless, hibernoma within a bone is rare and other conditions may demonstrate a similar imaging appearance. Interestingly, all reported cases of intraosseous hibernoma with CT or radiographic examination have demonstrated sclerosis.^1,12^ Our case is the first, to our knowledge, that was diagnosed exclusively on MRI without definite CT or radiographic correlation, although two cases were diagnosed from bone marrow aspirates without imaging and one was unspecified.^5,6,10^ Therefore, contrary to the concept of “intraosseous brown fat-associated sclerosis” proposed by Bonar et al^1^, we suggest broadening the differential diagnosis of intraosseous hibernoma to include non-sclerotic osseous lesions. It may be that our case was a developing hibernoma that had not yet elicited a sclerotic reaction, especially as our patient was much younger than the middle-aged and elderly patients more commonly reported. Cases of unusual imaging appearance such as ours require histopathological confirmation from bone biopsy to ensure appropriate diagnosis and treatment. Soft-tissue hibernoma arising from brown fat is a rare entity in itself and intraosseous hibernoma is even more rare, with only 14 cases documented previously.^1^

The exact aetiology and mechanism by which hibernomas occur is unclear. It appears that most cases are incidental and asymptomatic. Symptomatic presentation is more common where lesions compress adjacent anatomical structures such as nerve roots, although the aetiology of intraosseous hibernoma-related pain can be unclear. In our case, the aetiology of pain is difficult to establish in the absence of an expansile lesion but may be similar to that seen in bone metastases such as increased intraosseous pressure, periosteal stretching or microfracture.^13^ The pathology of intraosseous hibernoma in our case is consistent with that of others, suggesting a common pattern of benign, neoplastic proliferation of brown fat cells in a manner similar to more common, yet still infrequent, soft-tissue hibernomas.^9^ Since our biopsy sample was small and was obtained under CT guidance from an area where the lesion was occult, it could be argued that the specimen could reflect incidental residual brown fat in marrow adjacent to the lesion as opposed to a space-occupying mass composed of brown fat or hibernoma. The biopsy and ablation site on CT scan correspond to the initial lesion site on MRI, indicating that the original biopsy was obtained properly and represented a sample of the space-occupying mass, thereby supporting the diagnosis of intraosseous hibernoma.

Treatment of intraosseous hibernoma is not established owing to its rarity and generally asymptomatic, benign natural course. All of the five intraosseous hibernoma cases reported by Bonar et al^1^ were incidentally discovered on imaging studies for other pathologies such as cancer staging CT examinations. Only 2 of the 14 previously reported cases presented with back or buttock pain similar to our patient.^9,11^ In our case, the location of the lesion near the neural foramina may explain the symptomatic nature of the patient. The case reported by Botchu et al^9^ demonstrated a similar presentation with an initial lumbar spine MRI ordered for evaluation of low back and buttock pain, which demonstrated a lesion within the right ilium. Another case of sacral intraosseous hibernoma presented with lower back pain radiating down the ipsilateral foot.^11^ CT-guided radiofrequency ablation was performed in this case with complete pain relief at 9 months.^11^

Our case highlights the difficulties encountered in treating intraosseous hibernoma. No standardized treatment method exists, and symptomatic cases may benefit from ablation or surgical excision. Management approaches range from conservative methods of analgesic medication and steroid injections to ablation and surgical excision. In our case, microwave ablation with cementoplasty initially provided symptomatic relief, but then pain recurred with worsening symptoms, including paresthesias and numbness in a S3 distribution likely because of compressive effects of cementoplasty on the nerve root. Surgical removal of the bone cement and decompression of the S1–S3 nerve roots resulted in improvement of symptoms. Treatment should be tailored to the lesion location and symptomatic status of each patient on an individual basis.

## Learning points

1. Intraosseous hibernoma is a rare benign brown fat tumour that is generally asymptomatic but may cause symptoms owing to compressive effects on local structures.
2. A *T*_1_ weighted hypointense, *T*_2_ weighted hyperintense enhancing bone lesion may represent metastases, haemangioma, benign notochordal tumour or intraosseous hibernoma.
3. This case is unique as the lesion was occult on initial radiographs and CT scan, whereas most cases of intraosseous hibernoma are reported to be sclerotic.

## References

[1] Bonar SF, Watson G, Gragnaniello C, Seex K, Magnussen J, Earwaker J. Intraosseous hibernoma: characterization of five cases and literature review. Skeletal Radiol 2014; 43: 939–46. 24705581 10.1007/s00256-014-1868-8

[2] Yamaguchi T, Iwata J, Sugihara S, Mccarthy EF, Karita M, Murakami H, et al. Distinguishing benign notochordal cell tumors from vertebral chordoma. Skeletal Radiol 2008; 37: 291–9. 18188556 10.1007/s00256-007-0435-y PMC2257990

[3] Uglialoro AD, Beebe KS, Hameed M, Benevenia J. A rare case of intraosseous benign notochordal cell tumor of the coccyx. Orthopedics 2009; 32: 445–9. 19634813 10.3928/01477447-20090511-22

[4] Furlong MA, Fanburg-Smith JC, Miettinen M. The morphologic spectrum of hibernoma: a clinicopathologic study of 170 cases. Am J Surg Pathol 2001; J25: 809–14. 11395560 10.1097/00000478-200106000-00014

[5] Thorns C, Schardt C, Katenkamp D, Kähler C, Merz H, Feller AC. Hibernoma-like brown fat in the bone marrow: Jreport of a unique case. Virchows Arch J2008; 452: 343–5. 18188594 10.1007/s00428-007-0559-4

[6] Reyes AR, Irwin RB, Wilson JD, Desai HS Intraosseous hibernoma of the femur: an unusual case with a review of the literature. College of American Pathology Annual Meeting San Diego, CA; 2008.

[7] Kumar R, Deaver MT, Czerniak BA, Madewell JE. Intraosseous hibernoma. Skeletal Radiol 2011; 40: 641–5. 21207023 10.1007/s00256-010-1079-x

[8] Bai S, Mies C, Stephenson J, Zhang PJ. Intraosseous hibernoma: a potential mimic of metastatic carcinoma. Ann Diagn Pathol 2013; 17: 204–6. 22884657 10.1016/j.anndiagpath.2012.07.001

[9] Botchu R, Puls F, Hock YL, Davies AM, Wafa H, Grimer RJ, et al. Intraosseous hibernoma: a case report and review of the literature. Skeletal Radiol 2013; 42: 1003–5. 23474703 10.1007/s00256-013-1593-8

[10] Lynch DT, Dabney RS, Andrews JM Intraosseous hibernoma or Junusual location of brown fat?J J Hematopathol 2013; 6: 151–3.

[11] Imeen Ringe K, Rosenthal H, Länger F, Callies T, Wacker F, Raatschen HJ Radiofrequency ablation of a rare Jcase of an intraosseous hibernoma Jcausing therapy-refractory pain. J Vas Interv Radiol 2013; 24: 1754–6. 10.1016/j.jvir.2013.01.010 24160833

[12] Hafeez I, Shankman S, Michnovicz J, Vigorita VJ. Intra-osseous hibernoma: a case report and review of the literature. JSpine (Phila Pa 1976) 2015; E558–61. 25714847 10.1097/BRS.0000000000000851

[13] Mercadante S. Malignant bone pain: pathophysiology and treatment. Pain 1997; 69: 1–18. 9060007 10.1016/s0304-3959(96)03267-8

